# Exploring Gray Matter Alterations in Post‐Stroke Patients: A Structural Covariance Analysis

**DOI:** 10.1002/brb3.71667

**Published:** 2026-08-15

**Authors:** Juan‐Juan Lu, Xiang‐Xin Xing, Jiao Qu, Jia‐Jia Wu, Jie Ma, Mou‐Xiong Zheng, Xu‐Yun Hua, Jian‐Guang Xu

**Affiliations:** ^1^ Department of Rehabilitation Medicine, Yueyang Hospital of Integrated Traditional Chinese and Western Medicine Shanghai University of Traditional Chinese Medicine Shanghai China; ^2^ Department of Radiology Shanghai Songjiang District Central Hospital Shanghai China; ^3^ Department of Traumatology and Orthopedics, Shuguang Hospital Shanghai University of Traditional Chinese Medicine Shanghai China; ^4^ Department of Traumatology and Orthopedics, Yueyang Hospital of Integrated Traditional Chinese and Western Medicine Shanghai University of Traditional Chinese Medicine Shanghai China; ^5^ Engineering Research Center of Traditional Chinese Medicine Intelligent Rehabilitation Ministry of Education Shanghai China

**Keywords:** magnetic resonance imaging, source‐based morphometry, stroke, structural covariance network, volume

## Abstract

**Background:**

While structural covariance analysis is adept at assessing morphometric correlations among broadly distributed brain regions, limited research has focused on alterations in structural covariance networks post‐stroke. This study aims to identify significant brain gray matter changes and distinct structural covariance patterns in stroke patients.

**Methods:**

Nineteen post‐stroke patients (17 males, aged 50.79 ± 11.98 years) and 19 healthy controls (11 males, 44.32 ± 13.34 years) underwent structural magnetic resonance imaging. Source‐based morphometry (SBM) analysis was utilized to explore alterations in independent components representing gray matter structural networks. Between‐group comparisons were performed while controlling for age and gender. Structural covariance networks were constructed to explore changes in these patterns among stroke patients.

**Results:**

SBM analysis identified 13 independent components (ICs). Compared to controls, post‐stroke patients exhibited significant alterations in gray matter in the subcortical network (IC 4, IC 8, and IC 10). Additionally, in post‐stroke patients, there was a reduction in structural covariance between the subcortical‐cerebellar networks, accompanied by an increase between the subcortical‐prefrontal networks.

**Conclusion:**

The present findings highlight structural abnormalities within the subcortical network and significant alterations in the structural interactions between the subcortical, cerebellar, and prefrontal networks in stroke patients. The observed findings suggest large‐scale anatomical reorganization following stroke and provide insight into network‐level neuroanatomical alterations associated with post‐stroke functional deficits.

## Graphical abstract

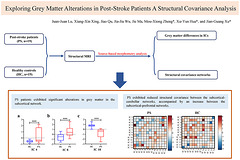

## 1 Introduction

Stroke is a leading cause of serious long‐term disability worldwide, characterized by high morbidity, mortality, and disability. Its complex pathophysiology has been extensively studied through advanced neuroimaging techniques. These studies have established a close association between post‐stroke deficits and structural damage at the lesion site, as well as disrupted structural and functional connectivity (Bonkhoff et al. 2022; Hope et al. 2017; Vicentini et al. 2021). Structural magnetic resonance imaging (sMRI) is commonly used to detail these post‐stroke structural changes, primarily focusing on gray matter (GM) alterations. Techniques such as voxel‐based morphometry (VBM) have been instrumental in demonstrating the reduction in gray matter volumes (GMV) in stroke patients and their correlations with motor, cognitive, and language functions (Sihvonen et al. 2020; Wang et al. 2018). Some researchers focused on abnormalities of cortical morphology with surface‐based morphology, and found a significant decrease in cortical thickness (CT), which was positively correlated with functional outcomes (Salah Khlif et al. 2022; Rojas Albert et al. 2022). However, the reporting of structural abnormalities in stroke has been inconsistent, influenced by the variability in the regions affected by specific functional deficits (Shi et al. 2017; Yang et al. 2018). Furthermore, the brain is recognized as an integrated functional and structural network. Traditional sMRI techniques like VBM and surface‐based morphology usually analyze isolated voxels or voxel groups, which can overlook the inter‐relationships between brain regions showing significant differences. This gap highlights the need for detailed investigation into the interactive relationships across different brain areas.

Structural covariance networks (SCNs) provide a framework for understanding these relationships by examining common morphological characteristics across brain regions (Geschwind 1965). Such covariance is thought to reflect shared trophic influences as well as common developmental and maturational processes among anatomically or axonally connected regions (Bernhardt et al. 2011). Previous research has typically employed seed‐based approaches to SCN analysis, selecting specific brain regions as seeds based on their GMV and CT to explore structural correlations within broader brain networks (Yan et al. 2022; Veldsman et al. 2020). Studies have shown enhanced structural interactions involving the ipsilesional primary motor cortex in subcortical stroke patients, indicating localized network reorganization (Chen et al. 2022; Wei et al. 2020). However, these approaches are inherently limited by prior region selection and may fail to capture whole‐brain patterns of structural reorganization. Given that post‐stroke deficits are increasingly recognized as network‐level dysfunctions rather than focal lesions, investigating structural covariance alterations may provide important insights into the neuroanatomical mechanisms underlying functional impairment and recovery.

Source‐based morphometry (SBM) offers a novel approach by utilizing independent component analysis (ICA) to identify brain networks characterized by covariation in gray matter concentration across multiple voxels (Xu et al. 2009). This method helps to circumvent the limitations of seed‐based approaches by capturing global patterns of structural covariation and has been successfully applied in the study of various cognitive and affective disorders (Qing et al. 2021; Sorella et al. 2019; Wang et al. 2022). Despite its advantages in capturing whole‐brain structural covariance patterns, SBM has been relatively underutilized in post‐stroke populations, where structural network reorganization remains insufficiently characterized. Moreover, previous studies have primarily focused on regional atrophy or seed‐based structural correlations, while alterations in whole‐brain gray matter structural covariance networks in post‐stroke patients remain insufficiently explored (Newman‐Norlund et al. 2024; Yang et al. 2019). To address this gap, the present study employs a data‐driven SBM approach to investigate alterations in whole‐brain gray matter structural covariance networks in post‐stroke patients.

In this study, we employed SBM to investigate gray matter alterations and structural covariance patterns associated with stroke. By comparing structural networks between post‐stroke patients and healthy controls, we aimed to characterize whole‐brain patterns of structural reorganization. We hypothesized that post‐stroke patients would exhibit significant alterations in gray matter networks, particularly within subcortical regions, along with disrupted structural covariance patterns involving subcortical, cerebellar, and prefrontal networks.

## 2 Material and Methods

### 2.1 Participants

This study involved 19 post‐stroke (PS) patients recruited from the outpatient of Yueyang Hospital of Integrated Traditional Chinese and Western Medicine, Shanghai University of Traditional Chinese Medicine. Additionally, 19 healthy controls (HC) were recruited from the local community within the same geographic area during the same study period. Formal matching on demographic characteristics was not performed during participant recruitment. All participants were aged between 18 and 70 years, right‐handed, and capable of undergoing the magnetic resonance imaging (MRI) examinations without gender restrictions. The patient group included individuals with a history of ischemic or hemorrhagic stroke occurring 3 to 12 months prior to the study (Shi et al. 2017; Yin et al. 2013). HCs underwent clinical interviews and MRI examinations to exclude neurological or psychiatric disorders, as well as structural brain abnormalities, including silent infarcts and other incidental findings. Exclusion criteria included the presence of other structural abnormalities on MRI, or any neurological or psychiatric diseases.

The study protocols were approved by the Institutional Ethics Committee of Yueyang Hospital of Integrated Traditional Chinese and Western Medicine, Shanghai University of Traditional Chinese Medicine (approval No.: KYSKSB2020‐116). All methods were carried out in accordance with the relevant guidelines and regulations stated in the Declaration of Helsinki. All participants provided written informed consents.

### 2.2 Magnetic Resonance Image Acquisition

Participants underwent T1‐weighted sMRI on a 3T MRI scanner (Siemens Healthcare, Erlangen, Germany). The scanning parameters included a repetition time (TR) of 1900 ms, echo time (TE) of 2.93 ms, inversion time of 900 ms, flip angle of 9°, field of view (FOV) of 256 mm× 256 mm, and voxel size = 1 × 1 × 1 mm^3^.

To facilitate the analysis of overlapping stroke lesions and subsequent data processing, T1‐weighted images from patients with right‐hemisphere stroke lesions were flipped from right to left along the midline prior to preprocessing (Miao et al. 2018). In all sMRI images, the left side was designated as the ipsilesional hemisphere and the right side as the contralesional hemisphere. Although this approach is commonly used in stroke neuroimaging studies to improve anatomical comparability across participants, it may reduce sensitivity to potential hemisphere‐specific structural effects.

### 2.3 Image Preprocessing

T1‐weighted sMRI images were preprocessed using Computational Anatomy Toolbox 12 (CAT12) (http://www.neuro.uni‐jena.de/cat/) in the Statistical Parametric Mapping 12 (SPM12) software package (https://www.fil.ion.ucl.ac.uk/spm/software/ spm12/), which was operated on Matlab R2013b (The Mathworks Inc., Natick, MA, USA). The CAT12 routine for VBM was employed to preprocess images, which encompassed several preprocessing steps: tissue segmentation [GM, white matter (WM), and cerebrospinal fluid (CSF)], normalization of images to the Montreal Neurological Institute (MNI) space using the Diffeomorphic Anatomic Registration Through Exponentiated Lie algebra (DARTEL) algorithm, and modulation to adjust voxel values to reflect gray matter volume accurately. Finally, the images were then smoothed using a Gaussian kernel with an 8 mm full width at half maximum (FWHM). These processed images were subsequently used for SBM analysis. The total intracranial volume (TIV) was the sum of all the GM, WM, and CSF volumes. No lesion masking, cost‐function masking, or enantiomorphic normalization procedures were applied. All normalized images were visually inspected to ensure satisfactory registration quality prior to subsequent analyses.

### 2.4 SBM Analysis

SBM is a data‐driven algorithm that utilizes ICA. The methodology for SBM in this study was modeled after the approach described by Xu et al. (2009). We employed the SBM module from the GroupICA v4.0c toolbox (http://icatb.sourceforge.net). In this process, the pre‐smoothed MR images of all participants were merged into a four‐dimensional (4D) data set. ICA decomposition was then performed on this aggregated dataset using the Infomax algorithm, selected for its robustness and widely demonstrated stability in neuroimaging applications. The number of components was set to 17, consistent with prior studies applying similar SBM/ICA frameworks in brain network decomposition (Kakeda et al. 2020), while acknowledging that model order selection remains a methodological consideration in data‐driven ICA approaches. To bolster the reliability of the independent components identified, stability analysis was performed using ICASSO approach (http://research.ics.aalto.fi/ica/icasso/), which included 20 iterations of the ICA. A quality Index (Iq) greater than 0.90 was considered indicative of a higher stable decomposition (Depping et al. 2016).

Each GMV image was converted into a 38‐row matrix corresponding to subjects, which was further divided into a mixing matrix and a source matrix. The mixing matrix consisted of rows representing subjects and columns representing components, with each element indicating the loading coefficient, the contribution of each component to the individual participant. Conversely, the source matrix was organized by component and voxel, depicted the spatial contribution of each component across voxels.

These matrices were pivotal for subsequent analysis: The mixing matrix facilitated between‐group comparisons, while the source matrix allowed for spatial visualization of the components. Each component's spatial map was converted into a three‐dimensional (3D) image, scaled to unit standard deviations (*Z*‐scores) and with a threshold set at *Z* > 3.5 and a minimum cluster size of 1 cm^3^ (Quidé et al. 2018). The component maps were then superimposed on an MNI‐normalized template, and stereotaxic coordinates were retrieved using the Talairach Daemon database (http://www.talairach.org/daemon.html).

### 2.5 SCN Analysis

Following the approach outlined in prior research (Qing et al. 2021), we used the loading coefficients from selected components to construct the SCN utilizing the Brain Covariance Connectivity Toolkit (BCCT) (https://github.com/JLhos‐fmri/BrainCovarianceConnectToolkitV2.1), operating on Matlab R2013b. Initially, SCNs were established separately for post‐stroke patients and healthy controls using partial correlation analysis, with age and gender included as covariates to control for potential confounding effects. Subsequently, we performed between‐group comparisons using the statistical module of BCCT, which included a robust permutation test (5000 permutations) to ensure the reliability of the results. A Bonferroni correction was applied to adjust for multiple comparisons, setting the threshold for statistical significance at *p* < 0.05.

### 2.6 Statistical Analysis

#### 2.6.1 Clinical Data

SPSS 25.0 software (SPSS, Chicago, IL, USA) was utilized to analyze the clinical data from all participants. Numerical variables, including age and TIV were presented as mean ± standard deviation (SD). We conducted two‐sample *t*‐tests to compare these variables between post‐stroke patients and healthy controls. Categorical variables, including gender and lesion laterality, were expressed as n (percentage). Differences in gender distribution between the two groups were assessed using the Chi‐Square (χ^2^) test. A *p* value of less than 0.05 was considered statistically significant.

#### 2.6.2 Between‐Group Differences

To evaluate significant differences between post‐stroke patients and healthy controls in component loading coefficients, ANOVA was performed using the SBM Stats module of the GroupICA v4.0c toolbox. Given the potential influence of demographic factors on brain morphology, both age and gender were included as covariates in the analysis. To account for multiple comparisons across the 13 independent components, Bonferroni correction was applied, with a corrected significance threshold of *p* < 0.0038 (0.05/13).

## 3 Results

### 3.1 Clinical Data Description

**FIGURE 1:**
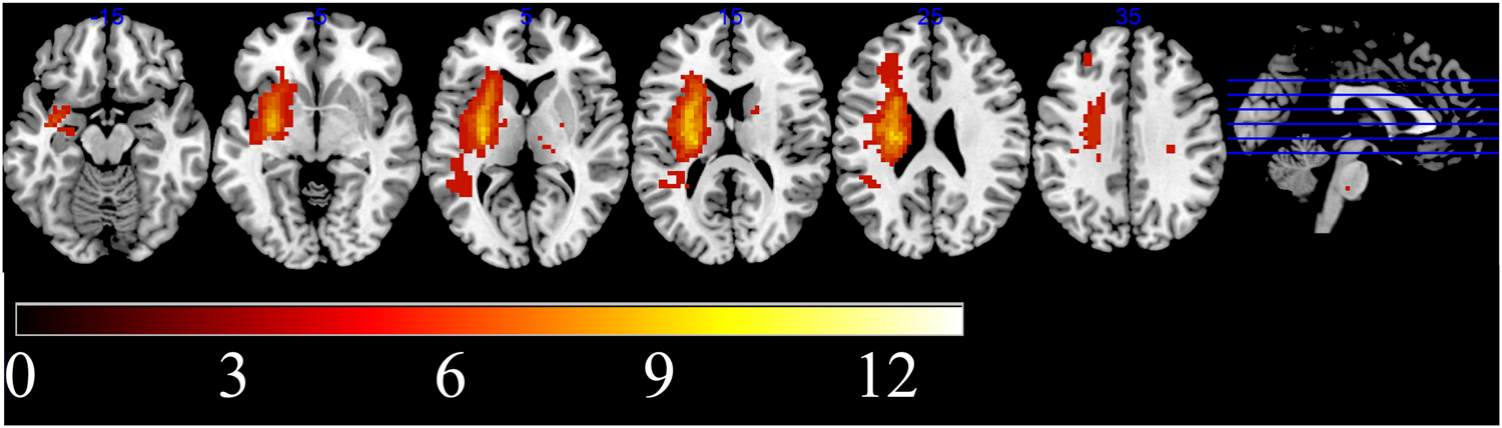
**Overlay map of lesions in post‐stroke patients**. An overlay map of all lesions observed in post‐stroke patients was displayed. To standardize the images for comparative analysis, we flipped lesion images from patients with right‐sided strokes from right to left along the midline. This adjustment allowed for the creation of a composite image representing lesion overlap across all patients. The color bar in the image indicates the density of lesion overlap, with warmer colors denoting higher concentrations of overlapping lesions.

In this study, we recruited 19 post‐stroke patients and 19 healthy controls. Their basic clinical characteristics are summarized in Table 1. We observed significant difference in gender between post‐stroke patients and healthy controls (*p* = 0.027). No significant difference was found in age or TIV between two groups. The overlay map of all patients' lesions is shown in Figure 1. The lesions were predominantly located in regions supplied by the middle cerebral artery and were classified as punctate (*n* = 7, lesion size: 0.46 ± 0.33 cm^3^) or territorial (*n* = 12, lesion size: 9.52 ± 7.16 cm^3^) (Jeon et al. 2018). Four patients exhibited bilateral stroke lesions; however, the larger stroke lesions size was predominantly unilateral, designating it as the ipsilesional side.

**TABLE 1:** Clinical characteristics of post‐stroke patients and healthy controls.

Characteristic	PS (*n* = 19)	HC (*n* = 19)	*p*‐value
Age (years)	50.79 ± 11.98	44.32 ± 13.34	0.124
Gender, male (%)	17 (89%)	11 (58%)	0.027
Lesion laterality, left (%)	10 (53 %)	/	/
Stroke type:			
Infarct (%)	14 (74 %)	/	/
Hemorrhage (%)	5 (26 %)	/	/
Time since stroke (months)	7.53 ± 3.47	/	/
TIV (ml)	1550.77 ± 130.66	1530.50 ± 164.25	0.676

*Note*: Numerical variables were presented as mean ± standard deviation (SD), and categorical variables as number (percentage).

Abbreviations: HC, healthy controls; PS, post‐stroke; TIV, total intracranial volume.

### 3.2 SBM Analysis

#### 3.2.1 Selection of Independent Components

**FIGURE 2:**
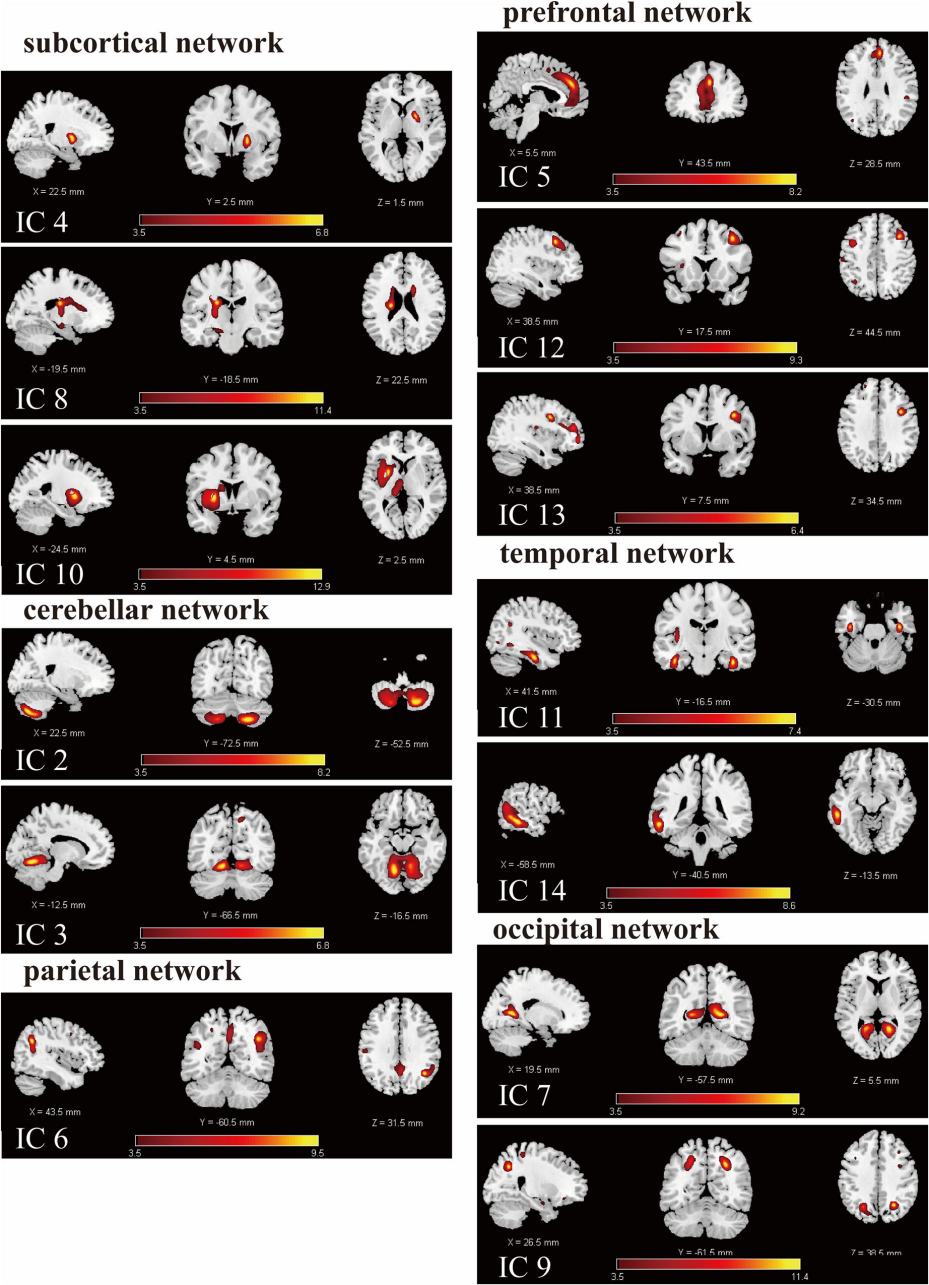
**Spatial maps of independent components identified by source‐based morphometry (SBM)**. Spatial maps of 13 structural components were presented, which were identified through SBM from 38 participants, including both post‐stroke patients and healthy controls. Displayed are the coronal, sagittal, and axial views of each component's spatial map, overlaid on the Montreal Neurological Institute (MNI) normalized template. The images are *Z*‐maps (*Z* > 3.5), and the color bar indicates the *Z*‐score, where higher values signify a greater contribution of voxels to the respective component. Abbreviations: ICs, independent components.

ICA was performed with a predefined model order of 17, resulting in 17 independent components (ICs). According to the criteria proposed by Xu et al. (2009), IC 1 was considered to be an artifact as most of the regions where this component primarily located do not contain GM. Additionally, ICs 15, 16, and 17 were considered unreliable due to their Iq being below 0.90, and were therefore excluded from this study. Table S1 summarizes the Iq for all retained components. The exclusion of these higher‐order components suggests that the selected model order may have exceeded the optimal dimensionality for the present dataset. Consequently, 13 ICs were selected for detailed analysis. As is shown in Figure 2, these ICs were primarily located in the subcortical, cerebellar, parietal, prefrontal, temporal, and occipital regions.

#### 3.2.2 Group Differences in Gray Matter Networks

**FIGURE 3:**
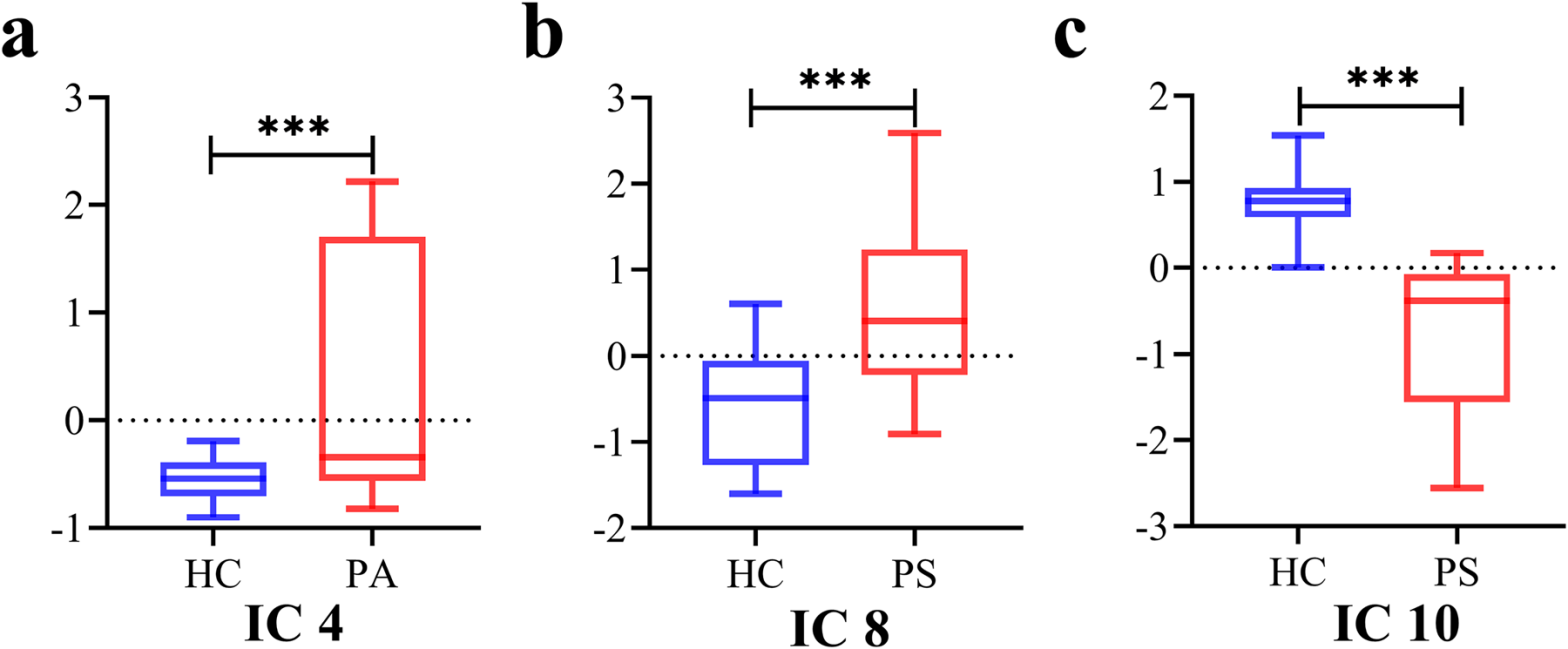
**Group difference in independent components between post‐stroke patients and healthy controls**. Significant differences in the loading coefficients of independent component (IC) 4 (a), 8 (b), and IC 10 (c) between post‐stroke (PS) patients and healthy controls (HC) were illustrated.

Among the 13 ICs, significant between‐group differences were identified in IC 4, IC 8, and IC 10 after controlling for age and gender. All components remained significant after Bonferroni correction for multiple comparisons (corrected threshold: *p* < 0.0038). Specifically, IC 4 [*F* (1, 36) = 15.637, *p* = 0.0003, Cohen's *d* = 1.32, 95% CI (0.57, 2.05)] and IC 8 [*F* (1, 36) = 14.960, *p* = 0.0004, Cohen's *d* = 1.29, 95% CI (0.55, 2.01)] exhibited larger loading coefficients in post‐stroke patients compared to healthy controls, involving right lentiform nucleus, bilateral extra‐nuclear areas. Conversely, IC 10 showed smaller loading coefficients in post‐stroke patients [*F* (1, 36) = 52.547, *p* < 0.0001, Cohen's *d* = 2.42, 95% CI (1.50, 3.32)], indicating brain atrophy mainly in the left lentiform nucleus, thalamus, extra‐nuclear, insula, and caudate (Figure 3, Table 2).

**TABLE 2:** Anatomical description of independent components 8 and 10.

Region	Brodmann area	Volume (cm^3^) left/right	Max *z*‐value for left/right hemisphere (Talairach coordinates *x*, *y*, *z*)
**IC 4**			
Sub‐gyral	/	0.1/0.9	4.2 (‐30, ‐49, 38)/5.9 (19, ‐52, 16)
**IC 8**			
Extra‐nuclear	/	3.3/1.9	11.4 (‐19, ‐16, 22)/7.5 (18, 11, 17)
Caudate	/	1.0/0.9	6.9 (‐16, ‐16, 24)/6.2 (15, 8, 18)
**IC 10**			
Lentiform nucleus	/	4.7/0.0	12.9 (‐24, 4, 3)/‐
Thalamus	/	5.6/0.0	12.8 (‐7, ‐17, 9)/‐
Extra‐nuclear	13, 47	5.9/0.0	11.0 (‐33, ‐5, ‐4)/‐
Insula	13	4.9/0.0	10.5 (‐36, ‐7, ‐2)/‐
Caudate	/	1.3/0.0	6.1 (‐13, 9, 12)/‐

*Note*: The *z*‐maps were thresholded at *z* > 3.5 and the anatomical regions were converted from Montreal Neurological Institute (MNI) coordinates to Talairach coordinates for the left (L) and right (R) hemispheres. The volume of voxels in each area is provided in cubic centimeters (cm^3^), summarizing only regions with positive contributions to the covariance and volume exceeding 1 cm^3^. Maximum *Z* values and their coordinate are listed, with anatomical descriptions sourced from the Talairach Daemon (http://www.talairach.org/daemon.html).

‐: no significant voxels.

### 3.3 Alterations in SCNs Between Post‐Stroke Patients and Healthy Controls

**FIGURE 4:**
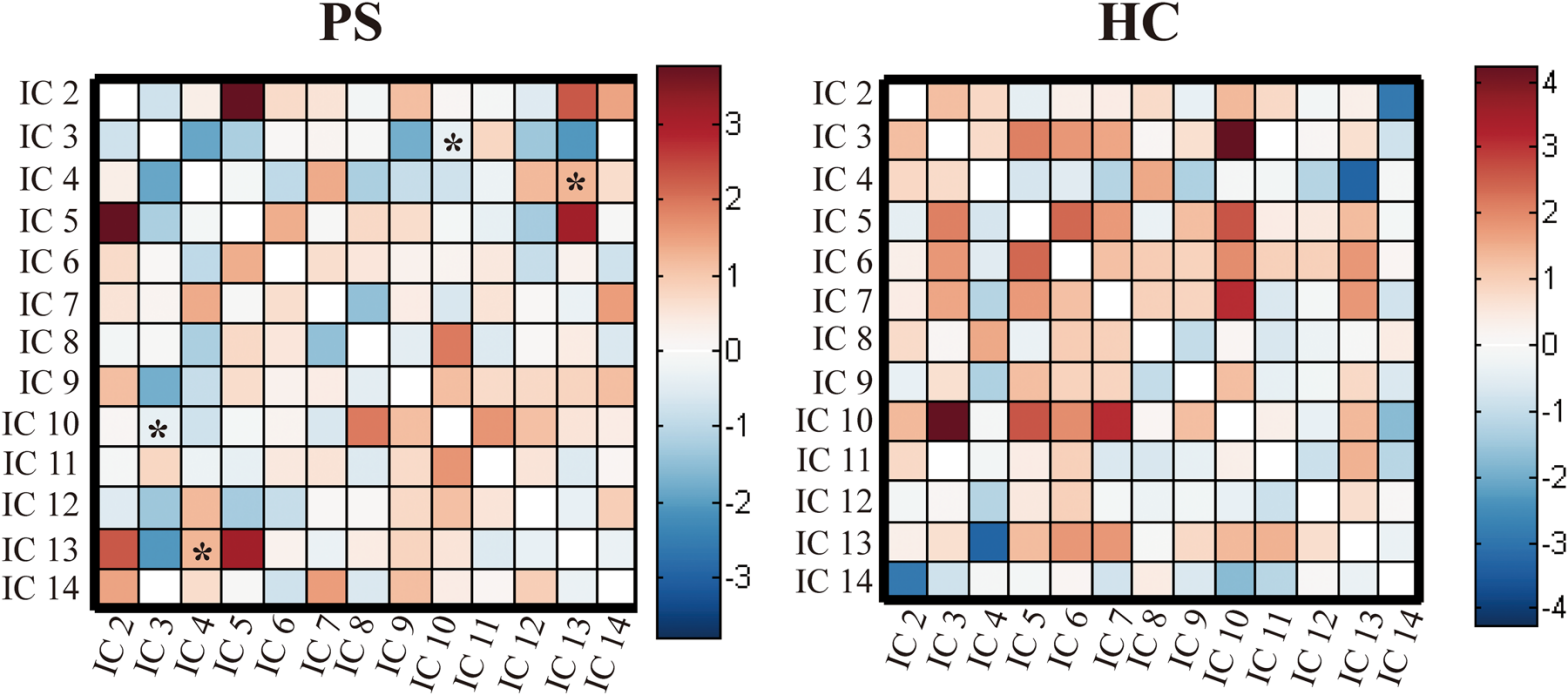
**Structural covariance among 13 selected components for post‐stroke patients and healthy controls**. The differences in the correlations of the components' *Z*‐scores across subjects for 13 selected components were shown. The color bar represents the intensity of averaged *Z*‐scores, with positive values indicating higher loading coefficients. * Statistical significance was determined using Bonferroni correction (*p* < 0.05). Abbreviations: PS, post‐stroke; HC, healthy controls.

Significant differences were observed in the SCNs of 13 selected components showed significant differences between post‐stroke patients and healthy controls (Figure 4). Specifically, there was a significantly lower structural covariance between IC 3 (mainly in cerebellar) and IC 10 (mainly in the left basal ganglia and thalamus) (raw covariance difference = ‐0.938, Cohen's *d* = 0.723, 95% CI: 0.028‐1.418), and a significantly higher structural covariance between IC 4 (mainly in the right lentiform nucleus) and IC 13 (mainly in the right middle/inferior/superior frontal gyrus) (raw covariance difference = 1.042, Cohen's *d* = 0.807, 95% CI: 0.102‐1.512) in post‐stroke patients compared to healthy controls. The anatomical details of independent components 3, 4 and 13 are reported in Table 3.

**TABLE 3:** Anatomical description of independent components 3, 4, and 13.

Regions	Brodmann area	Volume (cm^3^) left/right	Max *z* value for left/right hemisphere (Talairach coordinates *x*, *y*, *z*)
**IC 3**			
Declive	/	2.9/2.5	6.8 (‐12, ‐65, ‐11)/5.9 (15, ‐54, ‐11)
Culmen	/	5.0/5.6	6.7 (‐13, ‐60, ‐10)/6.1 (12, ‐57, ‐10)
**IC 4**			
Lentiform nucleus	/	0.0/2.2	‐/6.8 (22, 3, 1)
**IC 13**			
Middle frontal gyrus	6, 8, 9, 10, 46	0.9/5.5	4.8 (‐28, ‐2, 46)/6.4 (39, 9, 31)
Sub‐gyral	/	0.3/1.2	4.3 (‐24, 29, 33)/5.6 (42, 30, 16)
Inferior frontal gyrus	9, 46	0.0/1.6	‐/5.5 (43, 37, 13)
Insula	13	1.2/0.4	5.4 (‐36, ‐25, 18)/4.3 (40, ‐15, 16)
Superior frontal gyrus	6, 8, 9, 10	1.9/2.2	4.4 (‐21, 51, 26)/5.4 (28, 49, 17)

*Note*: The anatomical regions are summarized after thresholding the *Z*‐maps at *Z* > 3.5 and converting from Montreal Neurological Institute (MNI) coordinates to Talairach coordinates for the left (L) and right (R) hemispheres. The volume of voxels in each area is provided in cubic centimeters (cm^3^), and only regions with positive contributions to the covariance and volume exceeding 1 cm^3^ are included. Maximum *Z* values and their coordinates are listed, with anatomical descriptions sourced from the Talairach Daemon (http://www.talairach.org/daemon.html).

‐: no significant voxel.

## 4 Discussion

Using SBM, this study successfully extracted maximally spatially independent components from sMRI images, analyzing the variance of GMV across participants. We identified 13 distinct components, with three showing significant differences between groups after controlling for age and gender and correcting for multiple comparisons. Specifically, post‐stroke patients exhibited increased gray matter in IC 4 (contralesional lentiform nucleus), IC 8 (bilateral extra‐nuclear), and decreased gray matter in IC 10 (ipsilesional basal ganglia, thalamus, and insula). In addition, SCN analysis showed altered interactions, including decreased structural covariance between IC 3 (bilateral cerebellar) and IC 10, and increased structural covariance between IC 4 and IC 13 (contralesional frontal lobe). These patterns may suggest large‐scale anatomical reorganization following stroke, although the underlying biological significance of these alterations remains to be determined.

Gray matter injury has been getting extensive attention for the crucial roles of the gray matter in synaptic plasticity, cognitive, and motor functions. Post‐stroke gray matter volumetric changes can extend beyond perilesional areas to include contralesional hemisphere regardless of the stroke lesion site and affected hemisphere (Abela et al. 2015; Chen et al. 2021; Wei et al. 2020). In this study, we found decreased gray matter in the ipsilesional basal ganglia, thalamus, and insula in post‐stroke populations. Many of these brain regions showing gray matter loss were the lesioned regions, such as lentiform nucleus (putamen), caudate, and insula, possibly due to the disrupted blood supply (Wang et al. 2019). Such reduced volumes of the ipsilesional basal ganglia and thalamus were correlated with functional impairments of the affected hand (Ilves et al. 2022). Krishnamurthy et al. (2020) demonstrated factors affecting volumes of the ipsilesional thalamus, including intracortical volume, age, lesion volume, and age by time since stroke interactions. Interestingly, one longitudinal study divided patients with subcortical infarction into two groups by the affected side, and progressively reduced GMV in the ipsilesional thalamus was only observed in patients with left hemisphere stroke, which was greatly correlated with immediate recall function. Further analysis revealed that volumetric loss in thalamic subregions, which are connected to the left‐temporal, premotor, and prefrontal cortices, significantly contributed to impaired immediate recall. Therefore, it appears prudent to consider thalamus volumes when predicting verbal memory outcome, as targeted neuromodulation of specific thalamic subregions may effectively enhance verbal memory recovery. Additionally, damage to the insula could affect facial emotion recognition (van den Berg et al. 2021), apraxia of speech (Chenausky et al. 2020), and pharyngeal transit duration (Im et al. 2018) in post‐stroke patients. Previous studies involving both animals and humans have shown significant reductions in the GMV of the ipsilesional posterior insular cortex are also associated with central post‐stroke pain (Krause et al. 2016; Nagasaka et al. 2021).

We noted increased GM in the right lentiform nucleus of IC 4, left caudate of IC 8 and decreased GM in IC 10 in post‐stroke patients compared to healthy controls. This may be attributed to the capability of SBM analysis to discern volumetric alterations in specific regions within the same anatomical structure, recognizing smaller regions as independent components. These findings may suggest that different pathological processes following stroke affect different regions within the basal ganglia networks, potentially due to occlusions in different branches of the middle cerebral artery (Govaert et al. 2009; Kirton et al. 2008). This may result in varied volumetric changes across subcortical brain structures among subjects. Additionally, the identified ICs, such as IC 13, which is predominantly located in the contralesional frontal lobe and ipsilesional insula, illustrate that SBM analysis can detect patterns of common variation across multiple voxels and structures, thereby elucidating the complex spatial relationships between various brain regions. The reduction in gray matter in IC 10 may be related to stroke‐associated structural alterations, whereas the increased gray matter in IC 4 and IC 8 could potentially reflect adaptive structural changes. However, the cross‐sectional nature of the present study precludes definitive conclusions regarding the underlying mechanisms, and longitudinal studies incorporating functional assessments are needed to clarify their biological significance.

In this study, post‐stroke patients showed significant alterations in the structural covariance patterns among the selected ICs. The structural covariance analysis, which assesses covariation in gray matter morphology across brain regions and subjects, reveals inter‐regional coordination or synchronous effects within the brain network (DuPre and Spreng 2017). Specifically, significant changes were found between IC 3 (cerebellar network) and IC 10 (subcortical network), as well as between IC 4 (contralesional lentiform nucleus) and IC 13 (contralesional prefrontal network). These alterations extend beyond the ICs that displayed between‐group differences in GMV, indicating comprehensive anatomical reorganization at a whole‐brain network level, unaffected by lesion side and location of regional atrophy (Wang et al. 2019). Previous research using seed‐based structural covariance analysis has identified extensive covariant brain regions when utilizing significantly atrophic regions in bilateral posterior cerebellar lobe as seeds. These regions have been associated with behavioral deficits in ischemic pontine stroke (Wei et al. 2020). Additionally, changes in the structural covariance within the thalamocortical network have been linked to functional recovery of hand movements after ischemic stroke (Abela et al. 2015). Furthermore, reorganization within the prefrontal network may contribute to improve motor recovery following unilateral upper limb training (Xu et al. 2022). Collectively, the observed structural alterations may be consistent with multiple neurobiological processes involved in post‐stroke brain reorganization. Remote gray matter changes may be partially explained by diaschisis, whereby focal lesions disrupt functionally connected regions, leading to secondary structural alterations (Campos et al. 2023). In parallel, increased gray matter in specific regions could represent adaptive structural responses following stroke; however, their relationship to functional recovery cannot be determined from the present data. Furthermore, the altered structural covariance patterns observed in this study may be indicative of network‐level plasticity and widespread reconfiguration of large‐scale brain networks following stroke, rather than isolated regional changes.

There are several limitations in this study. Firstly, the relatively small sample size necessitates replication of these findings in larger cohorts to validate the conclusion. The limited sample size may have reduced statistical power and affected the stability of the structural covariance estimates. Therefore, the findings should be interpreted with caution. Secondly, the lack of restrictions on stroke etiology, lesion locations, affected hemispheres, lesion characteristics, and post‐stroke duration might have introduced heterogeneity, making it difficult to attribute the observed structural covariance alterations to specific pathophysiological mechanisms or stroke subtypes. Although image registration was visually inspected and considered satisfactory, tissue misclassification around lesioned regions cannot be completely excluded and may have influenced component estimation. Thirdly, the absence of functional evaluations precluded direct evaluation of the relationships between structural alterations and clinical outcomes. Future studies integrating behavioral and functional measures are needed to clarify the functional significance of the observed network changes. Fourthly, the predefined model order used in ICA‐based SBM may have influenced component estimation. Future studies may benefit from data‐driven dimensionality determination and latent‐variable approaches. Moreover, ICA‐derived components may retain error‐related variance within the resulting structural covariance networks. Finally, residual confounding related to the unequal gender distribution between groups cannot be completely excluded, despite adjustment for gender in the statistical analyses.

## 5 Conclusions

This study effectively utilized SBM to identify ICs representing gray matter networks, revealing significant volumetric differences between post‐stroke patients and healthy controls, particularly within the subcortical network. We also observed significant alterations in the structural covariance patterns, especially between the subcortical network and cerebellar and prefrontal networks in post‐stroke patients. These findings indicate a pattern of large‐scale network reorganization following stroke. While the functional implications of these structural covariance changes remain speculative in the absence of behavioral data, they may provide a basis for future hypotheses regarding motor, cognitive, and emotional processes. Overall, this study contributes to a better understanding of the network‐level neuroanatomical alterations associated with stroke and may inform future research on targeted interventions.

## Author Contributions

**Jie Ma**: methodology, funding acquisition. **Juan‐Juan Lu**: writing – original draft, visualization, formal analysis. **Jiao Qu**: data curation, investigation. **Xiang‐Xin Xing**: data curation, investigation, validation. **Mou‐Xiong Zheng**: conceptualization, writing – review and editing, methodology, funding acquisition. **Jian‐Guang Xu**: conceptualization, supervision, project administration, funding acquisition. **Jia‐Jia Wu**: methodology, funding acquisition. **Xu‐Yun Hua**: conceptualization, supervision, funding acquisition, writing – review and editing.

## Funding

This work was supported by the National Natural Science Foundation of China (grant numbers: 82272583, 82172554, 82272589, 82302870); National Key R&D Program of China (grant numbers: 2018YFC2001600, 2018YFC2001604); Shanghai Health Care Commission (grant number: 2022JC026); Shanghai Science and Technology Committee (grant number: 22010504200); Shanghai Rising‐Star Program (grant number: 23QA1409200); Shanghai Youth Top Talent Development Plan; Shanghai “Rising Stars of Medical Talent”—Distinguished Young Medical Talent Program; Shanghai Talent Development Fund (grant number: 2021074); High‐level Chinese Medicine Key Discipline Construction Project (Integrative Chinese and Western Medicine Clinic) of National Administration of TCM (grant number: zyyzdxk‐2023065); Science & Technology Development Fund of Shanghai University of Traditional Chinese Medicine (grant number: 23KFL112).

## Ethics Statement

The study protocols were approved by the Institutional Ethics Committee of Yueyang Hospital of Integrated Traditional Chinese and Western Medicine, Shanghai University of Traditional Chinese Medicine (approval No.: KYSKSB2020‐116).

## Conflicts of Interest

The authors declare that they have no conflicts of interest.

## Supporting information




**Table S1**: brb371667‐sup‐0001‐TableS1.docx

## Data Availability

The data supporting this study's findings are available from the corresponding author upon reasonable request.
