# Supplementary material for: Exploring Gray Matter Alterations in Post‐Stroke Patients: A Structural Covariance Analysis
Source: Brain Behav. 2026 Aug 15;16(8):e71667. doi: 10.1002/brb3.71667 (PMC13477228; doi:10.1002/brb3.71667)
Supplement: Supplementary file 1 [file BRB3-16-e71667-s001.docx]

**Supplementary Information**

This supplementary document provides detailed information about the Iq for all retained components.

**Table S1 ICASSO stability indices (Iq values) for retained independent components.**

| **Independent Component** | **Iq** |
| --- | --- |
| IC 2 | 0.979 |
| IC 3 | 0.970 |
| IC 4 | 0.982 |
| IC 5 | 0.972 |
| IC 6 | 0.969 |
| IC 7 | 0.965 |
| IC 8 | 0.967 |
| IC 9 | 0.952 |
| IC 10 | 0.956 |
| IC 11 | 0.914 |
| IC 12 | 0.932 |
| IC 13 | 0.915 |
| IC 14 | 0.934 |

Iq values were derived from ICASSO analyses. Components with Iq > 0.90 were considered stable and retained for subsequent analyses.
